# LRP4 is required for the olfactory association task in the piriform cortex

**DOI:** 10.1186/s13578-022-00792-9

**Published:** 2022-05-07

**Authors:** Min Yan, Mingtao Xiong, Yongqiang Wu, Dong Lin, Peng Chen, Jiang Chen, Ziyang Liu, Hang Zhang, Dongyan Ren, Erkang Fei, Xinsheng Lai, Suqi Zou, Shunqi Wang

**Affiliations:** 1 Laboratory of Synaptic Development and Plasticity, Institute of Life Science and School of Life Sciences, Nanchang University, Nanchang, 330031 Jiangxi China; 2 School of Basic Medical Science, Nanchang University, Nanchang, 330031 Jiangxi China; 3 Queen Mary School, Nanchang University, Nanchang, 330031 Jiangxi China; 4 Mental Health Center, West China Hospital of Sichuan University, Chengdu, 610041 Sichuan China

**Keywords:** LRP4, Piriform cortex, Olfactory function, Golgi staining, Spine density

## Abstract

**Background:**

Low-density lipoprotein receptor-related protein 4 (LRP4) plays a critical role in the central nervous system (CNS), including hippocampal synaptic plasticity, maintenance of excitatory synaptic transmission, fear regulation, as well as long-term potentiation (LTP).

**Results:**

In this study, we found that *Lrp4* was highly expressed in layer II of the piriform cortex. Both body weight and brain weight decreased in *Lrp4*^*ECD/ECD*^ mice without TMD (Transmembrane domain) and ICD (intracellular domain) of LRP4. However, in the piriform cortical neurons of *Lrp4*^*ECD/ECD*^ mice, the spine density increased, and the frequency of both mEPSC (miniature excitatory postsynaptic current) and sEPSC (spontaneous excitatory postsynaptic current) was enhanced. Intriguingly, finding food in the buried food-seeking test was prolonged in both *Lrp4*^*ECD/ECD*^ mice and *Lrp4* cKO (conditional knockout of Lrp4 in the piriform cortex) mice.

**Conclusions:**

This study indicated that the full length of LRP4 in the piriform cortex was necessary for maintaining synaptic plasticity and the integrity of olfactory function.

**Supplementary Information:**

The online version contains supplementary material available at 10.1186/s13578-022-00792-9.

## Introduction

LRP4 plays an essential role in synaptic plasticity and excitatory transmission in the CNS, and it is expressed in the hippocampus, olfactory bulb, cerebellum, and neocortex [1–3]. Recently, Zhang et al*.* found that genetic deletion of *Lrp4* increased Aβ plaques formation in Alzheimer’s disease (AD) mice and exacerbated the deficits in neurotransmission, cognition, and synchrony between the hippocampus and prefrontal cortex [4]. Astrocytic LRP4 played a crucial role in AD pathology and cognitive function. Sun et al*.* found that astrocytic LRP4 regulates ATP release [1]. Glutamate release of the hippocampal neurons was impaired because of ATP release enhancement in *Lrp4* knockout astrocytes [1]. Recent research shows that LRP4 plays a significant role, including hippocampal synaptic plasticity, excitatory synaptic transmission, fear regulation, and LTP [5–7].

LRP4 is a member of the low-density lipoprotein receptor (LDLR) family. As a single transmembrane protein, LRP4 contains a short ICD and a large extracellular domain (ECD), possessing eight LDLa repeats, six EGF repeats, and four β-propeller domains [8–13]. Being a receptor of Agrin, LRP4 is critical for MuSK activation, AChR clustering in the neuromuscular junction (NMJ) formation, and NMJ maintenance [5, 8–10, 13]. Biochemical studies confirm that LRP4 is a crucial protein in the complex with Agrin and MuSK, and importantly, ECD of LRP4 is the direct interaction site among them [5, 10, 12–14]. Though *Lrp4* null is perinatally lethal [9, 15, 16], ECD of LRP4 may function as a scavenger for signal modulators or signaling ligands in the extracellular space, consequently maintaining critical signaling thresholds for development [17].

This research found that *Lrp4* was supremely expressed in layer II of the piriform cortex, besides the hippocampus in the CNS in the previous report [1]. To explore whether the full length of LRP4 in the piriform cortex involves the sense of smell, we investigated *Lrp4*^*ECD/ECD*^ mice and *Lrp4* cKO mice. The body and brain weight of *Lrp4*^*ECD/ECD*^ mice decreased. Intriguingly, finding food in the buried food-seeking test was prolonged in both types of mice, implying the olfactory function was impaired. In the piriform cortical neurons of *Lrp4*^*ECD/ECD*^ mice, the dendritic spine density increased, and the frequency of both sEPSC and mEPSC was enhanced. These data indicated that the full length of LRP4 in the piriform cortex was necessary to maintain synaptic plasticity and the integrity of olfactory function.

## Materials and methods

### Animals

*Lrp4*^*LacZ/*+^ mice were described before; in brief words, β-galactosidase (β-gal) protein cassette, including stop code and a polyadenylation termination signal, was inserted into the downstream of *Lrp4* promoter [1]. *Lrp4*^*ECD/ECD*^ mice (JAX stock #013157) were described before, which introduced a stop codon just upstream of TMD of *Lrp4* [6, 7, 18]. CAG-*Cas9* mice (C57BL/6-*Gt(ROSA) 26Sor*^*tm1*(CAG−*Cas9*)Smoc^, NM-KI-00120, Shanghai Model Organisms Center) was a gift from Dr. Dongmin Yin (East China Normal University). Mice were housed in a 12-h light/dark cycle room, 23–25 °C, with adlibitum access to rodent chow diet and water. Experiments involving animals were conducted according to the “guidelines for the care and use of experimental animals” issued by Nanchang University, following the directive 2010/63/EU to protect animals used for scientific purposes. For in vivo experiment, surgery was executed with sodium pentobarbital anesthesia (50 mg/kg, ip injection), and all efforts were made to minimize suffering [19]. Male mice were utilized for the experiments, and after terminal experiments, the mice were euthanized by carbon dioxide inhalation.

### Western blotting

Western blotting was performed as described previously [20] with minor modifications. In brief, total proteins were extracted by RIPA buffer (150 mM NaCl, 1.0% NP-40, 0.5% Na-deoxycholate, 0.1% SDS, 50 mM pH 8.0 Tris), supplementary with phenyl-methane sulfonyl fluoride (PMSF) and proteinase inhibitor mix before using. After electrophoresis, samples were transferred to the PVDF membrane (Millipore, USA) with transfer buffer (25 mM Tris, 192 mM Glycine, 20%(v/v) Methanol). The membrane was blocked by blocking buffer(5%(m/v) Skim-milk, 20 mM Tris, 150 mM NaCl, 0.1%(v/v) Tween20) for 2 h and was washed 3 times with washing buffer (20 mM Tris, 150 mM NaCl, 0.1%(v/v) Tween20). Anti-LRP4 (Rabbit-anti-mouse, Lab produced, 1:1000), anti-GAPDH (Mouse monoclonal, ab8245, Abcam, 1:2000) or anti-α-tubulin (Mouse monoclonal, SC-23948, Santa Cruz Biotechnology, 1:1000) primary antibody was added and incubated at 4 °C overnight. The HRP-labeled secondary antibody (Goat anti-Mouse IgG 31431, Goat anti-Rabbit IgG, 31466, Thermo Fisher Scientific, 1:2000) was added to incubate at room temperature for 2 h and then washed three times. LuminataTM Crescendo Western HRP Substrate was added. Immunoreacted bands were captured by an enhanced chemiluminescence system (BIO-RAD, USA).

### Quantitative real-time PCR (qPCR)

Total RNA was isolated from mice brain tissues according to the manufacturer’s instructions of TRIzol Reagent (Invitrogen), and complementary DNA (cDNA) was synthesized following the manufacturer’s protocol of High-Capacity cDNA Reverse Transcription Kit (Thermo Fisher Scientific, 4368814). The qPCR primer sets as below: *Lrp4* (5′-GTGTGGCAGAACCTTGACAGTC-3′ and 5′-TACGGTCTGAGCCATCCATTCC-3′), and *Gapdh* (5′-CATCACTGCCACCCAGAAGACTG-3′ and 5′-ATGCCAGTGAGCT TCCCGTTCAG-3′). qPCR was carried out by the Step One Plus Real-Time PCR system (Applied Biosystems) using the mix. mRNA expression levels were normalized to the reference gene *Gapdh* using the ΔCT method [21, 22].

### Open-field test

In behavioral tests, the activity levels of the mice were evaluated at P50. The open field (40 × 40 × 20 cm) measured the mice’s moving distance over 10 min. A video camera recorded the data, and the data were analyzed using the behavior analysis software ANY-maze (Stoelting Co., Wood Dale, IL, USA).

### Buried food-seeking test

Mice were food-deprived for 2 days, trained for 2 days, and tested continuously 3 days, with ad libitum access to enough water. Food was randomly placed in the box and was buried under padding for 0.5 cm in testing trials. The mice seizing the food with their front paws and biting were regarded as finding the food. The time was recorded when the mice were placed in the container and found the food.

### X-gal staining

X-gal (5-bromo-4-chloro-3-indolyl-beta-d-galacto-pyranoside), the inert chromogenic substrate for β-gal, hydrolyzes X-gal into colorless galactose and 4-chloro-3-brom-indigo, forming an intense blue precipitate. Mice brains were fixed for 8–10 h in 2% (m/v) paraformaldehyde (PFA) at room temperature and then were transferred into 30% sucrose solution at 4 °C. The brain slices were added PBS (phosphate-buffered sodium, pH 7.4) in a wet box, washing the slices at room temperature with PBS. After rinsing for 10 min, adding dye solution, put the slices at 37 °C for 8 h. After the reaction, brain slices were washed three times with PBS.

### Immunohistochemistry co-staining with X-gal

X-gal-stained brain slices were immersed in blocking solution (10% (v/v) donkey serum, 1% (m/v) calf serum albumin, 0.5% (v/v) Triton X-100 in PBS) for 2 h. Then the slices were rinsed with PBS at room temperature. Incubating the brain slices with the primary antibody (Rabbit anti-GFAP antibody, Z0334, Dako,1:1000) at 4 °C overnight. The slices were incubated with a secondary antibody (Alexa Fluor^®^ 488 Goat anti-rabbit, A32731TR, Thermo Fisher Scientific, 1:1000) for 2 h. Brain slices were washed with PBS, and then the images were captured by a microscope (Olympus FSX100).

### Nuclear fast red counterstaining

Put the X-gal-stained or co-stained brain slices into a vitreous tank containing nuclear fast red staining solution for 5 min. The slices were put into glass tanks containing 50%, 75%, and 90% ethanol in sequence, each for 4 min. The slides were transferred into 100% ethanol two times. Then the slides were put into xylene for 5 min. At last, the slides were sealed with Hydro mount (National Diagnostics). Images were captured by an inverted fluorescence microscope (Olympus FSX100).

### Virus packaging and stereotactic injection (*Lrp4* cKO mice)

The AAV-PHP capsids engineering was performed as previously described [23, 24]. pAAV-gRNA-CMV-mCherry contains the rAAV genome of interest, pUCmini-iCAP-PHP encodes the viral capsid and replication proteins, and pHelper encodes adenoviral proteins necessary for replication. Using the triple-transfection (pAAV-gRNA-CMV-mCherry:pUCmini-iCAP-PHP:pHelper = 5:2:1) with polyethylenimine (PEI), a single-stranded rAAV genome is packaged into an AAV-PHP capsid in HEK293 cells. In brief, *Lrp4* gRNA (GTACCTGTATCCCCGCCCAGTGG) was inserted into pAAV-gRNA-CMV-mCherry to produce AAV-*Lrp4* gRNA, and the control virus AAV-vector containing pAAV-gRNA-CMV-mCherry vector. Five days after triple-transfection, the AAV (AAV-*Lrp4* gRNA or AAV-vector) was performed with harvest, purification, and titration test. CAG-*Cas9* mice (6 weeks) were then anesthetized with 1% pentobarbital sodium (100 mg/kg, i.p.). Select the middle position of the brain, cut off the mouse’s topcoat, disinfect with alcohol, cut the scalp longitudinally, and separate the skin with hemostatic forceps, after the periosteum was removed, the three-dimensional coordinates (X = ± 3.75; Y, bregma = − 1.2; Z, depth = − 5.48) were read by brain locator, and 0.2 μl AAV suspension liquid was injected into the piriform cortex. After 21 days, the mice were sacrificed, and their brains were harvested quickly and stored at − 20 °C for immunofluorescent staining.

### Immunofluorescent staining

The brain slices were rinsed with PBS at room temperature and were immersed with antibody blocking solution (0.5% (v/v) Triton X-100,10% (v/v) donkey serum,1% (m/v) calf serum albumin, in PBS) at room temperature for 2 h. And then, the slices were rinsed with PBS at room temperature. The slices were incubated with primary antibody anti-NeuN (mouse monoclonal, MAB377, Merck Millipore, 1:1000) at 4 °C overnight. The slices were washed with PBS at room temperature three times for 10 min. The secondary antibody (Alexa Fluor^®^ 568 Goat anti-Mouse A-11019, Thermo Fisher Scientific, 1:1000) was added, and then the slices were incubated at room temperature for 2 h in the dark. After washing with PBS, samples were mounted in a Hydro mount (National Diagnostics).

### Golgi staining

Golgi staining was performed following the FD Rapid Golgi Stain™ Kit (FD NeuroTechnologies, PK-401, USA). Staining solution D and solution E were mixed with ultra-pure water in a ratio of 1:1:2. Dying at room temperature for 10 min. Slides with the slices were washed in ultra-pure water twice, then put into the plate hole containing 50%, 75%, 90%, and 100% ethanol in sequence, each for 4 min. After dehydration 3 times, the slides were put into xylene for 1 h and mounted in Hydro mount (National Diagnostics). Images were captured by an Olympus fluorescence microscope (FSX100), and dendritic spines were counted with image J.

### Electrophysiological recording

The electrophysiological recording was performed as previously described [25, 26]. Brain sections of 300 μm were cut with a vibratome (Leica, VT1000S) in oxygenated (95% O_2_, 5% CO_2_) sectioning buffer (120 mM Choline-Cl, 2.5 mM KCl, 0.5 mM CaCl_2_, 7 mM MgCl_2_, 1.25 mM NaH_2_PO_4_, 26 mM NaHCO_3_, and 25 mM glucose) at 4 °C. Slices were then placed into the oxygenated artificial cerebrospinal fluid (ACSF) (124 mM NaCl, 2.5 mM KCl, 2.5 mM CaCl_2_, 2 mM MgSO_4_, 1.25 mM NaH_2_PO_4_, 26 mM NaHCO_3_, and 10 mM glucose) at 34 °C for 30 min and recovery at room temperature (25 ± 1 °C) for more than 1 h before recording. Slices were transferred to a recording chamber under perfusion ACSF (2 ml/min, 32–34 °C). Pyramidal neurons in the piriform cortex were visualized with infrared optics using an upright fixed microscope equipped with a 40× water-immersion lens (FN1, Nikon) and CCD monochrome video camera (IR-1000, DAGE-MTI). Patch pipettes (resistance of 3–5 MΩ) were prepared by a horizontal pipette puller (P-1000; Sutter Instruments). For sEPSC recording, pyramidal neurons were held at − 70 mV in the present of 20 µM bicuculline methiodide (BMI), with the pipette solution (125 mM K-gluconate, 5 mM KCl, 10 mM HEPES, 0.2 mM EGTA, 1 mM MgCl_2_, 4 mM Mg-ATP, 0.3 mM Na-GTP and 10 mM phosphocreatine, pH 7.35). For mEPSC recording, 20 μM BMI and 1 μM TTX were added into the perfusion ASCF to block GABA receptor-mediated currents and action potentials.

### Statistical analysis

**Fig. 1:**
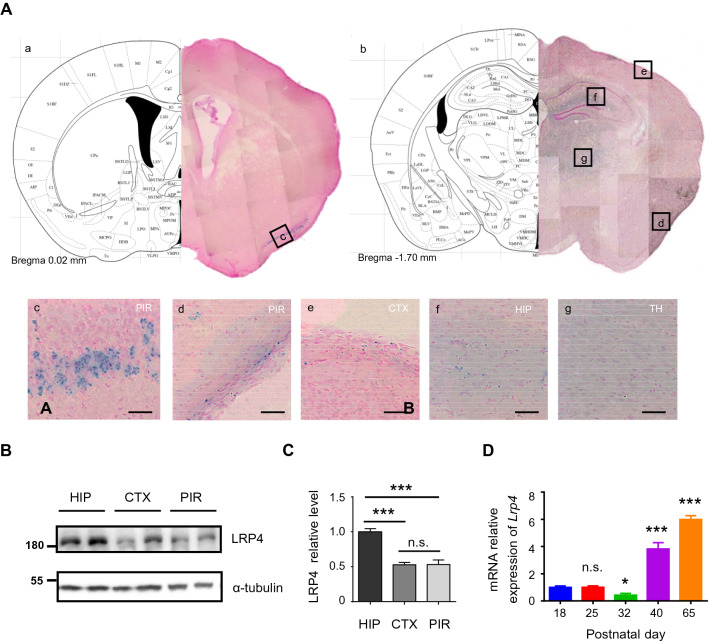
Highly expression of *Lrp4* in piriform cortex. **A** X-gal staining of *Lrp4*^+*/Lac*^ mice brain slice. PIR, piriform cortex; CTX, cerebral cortex; HIP, hippocampus; TH, thalamus; *Lrp4*^+*/Lac*^ mice, n = 5. **B** *Lrp4* expression in wild-type adult mice brain was confirmed by Western blotting (Wild type mice, n = 6). **C** Quantification of LRP4 protein level relative to α-tubulin from Western blotting. LRP4 relative level in the HIP was normalized to a value of 1. **D** mRNA expression of *Lrp4* in piriform cortex in postnatal wild-type mice. *Lrp4* expression values were calculated relative to *Gapdh* by using the 2^−ΔCT^ methods, and the P18 group was set to a value of 1. (Wild type mice per group, n ≥ 5; RNA pool was made for qPCR in each group; Values were means ± SEM, one-way ANOVA with multiple comparisons, compared other group with P18 group. n.s. no significant, * P < 0.05, *** P < 0.001)

Data were statistically analyzed with GraphPad Prism 6.0 software systems, and the values were expressed as means ± standard error (means ± SEM). One-way ANOVA (Fig. 1C), two-way ANOVA (Fig S1B, 5E, 6 J), and t-test (Other figures) analyzed the normality distributed data. All tests were two-sided. * p < 0.05, ** p < 0.01, *** p < 0.001.

## Results

### Expression of *Lrp4* in the piriform cortex

*Lrp4*^*LacZ/*+^ mice were utilized to locate the *Lrp4* expression region by X-gal staining because X-gal is the substrate for β-gal. And X-gal staining results showed that *Lrp4* was expressed in many brain regions, such as the piriform cortex, hippocampus, and cerebral cortex (Fig. 1A). To verify LRP4 protein relative expression in the brain regions, we conducted western blotting experiments (Fig. 1B). LRP4 relative level in the hippocampus is high, and LRP4 relative level is not different between the piriform cortex and in the cerebral cortex (Fig. 1C). To detect the expression profile of *Lrp4* in the piriform cortex, we also used quantitative fluorescence PCR to quantify the expression of *Lrp4* in the piriform cortex in postnatal wild-type mice (Fig. 1D). *Lrp4* was at a low and stable level in adolescence and became highly expressed in adulthood in the piriform cortex, suggesting that *Lrp4* expression was related to the development.

### High expression of *Lrp4* in layer II of the adult piriform cortex

**Fig. 2:**
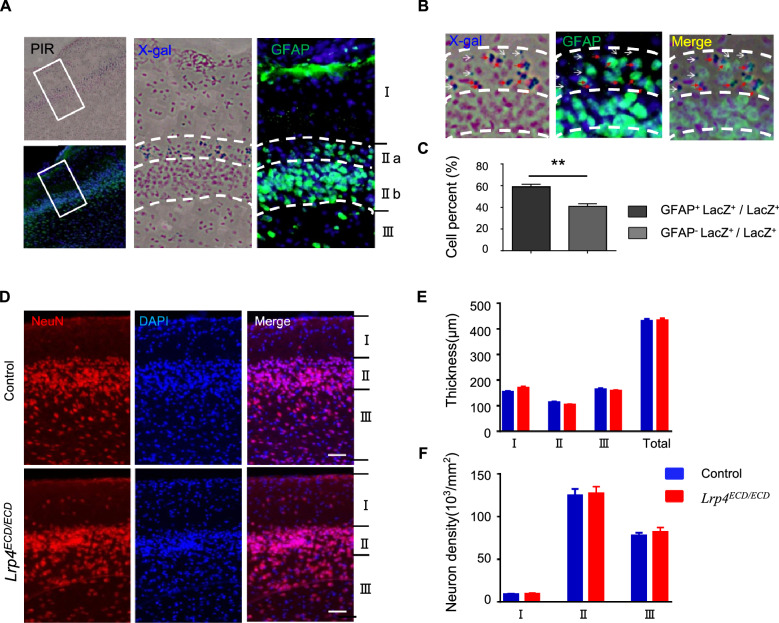
*Lrp4* was expressed in the layer II of the piriform cortex. **A** *Lrp4*^+*/Lac*^ mice brain slice was co-stained with X-gal and anti-GFAP. GFAP positive cells were mainly distributed in the second superficial area of the piriform cortex, and X-gal staining positive cells were primarily located in layer IIa of the piriform cortex; nuclei were counterstained fast red. **B** Layer II of the piriform cortex. X-gal co-stained with GFAP^+^ cells (red arrow) and GFAP^−^ cells (white arrow; *Lrp4*^+*/Lac*^ mice, n = 5). **C** Percent of GFAP^+^ and GFAP^−^ cells in LacZ^+^ cell population in Layer IIa of the piriform cortex (*Lrp4*^+*/Lac*^ mice n = 3). **D** Representative images of neuron staining (anti-NeuN) of the two types of mice brain. **E** The thickness of the piriform cortex in layers I, II, and III did not change in *Lrp4*^*ECD/ECD*^ mice. **F** The neuronal density was not different in the three layers (Mice per type, n = 7. Scale bar = 50 μm. Values were means ± SEM. **P < 0.01)

Taking advantage of *Lrp4*^*LacZ/*+^ mice, we used immunohistochemical co-staining to identify the location of LRP4. The result indicated that LRP4 was mainly expressed in layer II of the piriform cortex (Fig. 2A). The co-staining assay results suggested that X-gal co-stained with anti-GFAP (astrocyte and neuron stem cell marker) and GFAP-negative cells (Fig. 2B). In LacZ positive cell population, the percent of GFAP positive (GFAP^+^ LacZ^+^) cells was higher than the percent of GFAP negative (GFAP^−^ LacZ^+^) cells (Fig. 2C).

### Normal structure of the piriform cortex in *Lrp4*^*ECD/ECD*^ mice

ECD of LRP4 could maintain critical signaling thresholds for development [17]. Therefore, *Lrp4*^*ECD/ECD*^ mice could develop much better than *Lrp4* null mice because the latter mice are dead at birth [9, 15, 16]. The body and brain weight of *Lrp4*^*ECD/ECD*^ mice were lighter than the control mice (Additional file 1: Fig S1A–D). *Lrp4*^*ECD/ECD*^ mice showed typical tight-knit morphology (Additional file 1: Fig S1E).

It was unclear whether the morphology of the piriform cortex in *Lrp4*^*ECD/ECD*^ mice changed. Immunofluorescent staining was carried out to observe the piriform cortex of the mice. There was no remarkable difference in the thickness of *Lrp4*^*ECD/ECD*^ mice compared with the control mice (Fig. 2D, E), and the piriform cortical neuron density was similar in the two types of mice (Fig. 2F). We speculated that the ECD of LRP4 maintained the typical structure of the piriform cortex.

### Increase of neuronal mature spine density in *Lrp4*^*ECD/ECD*^ mice

**Fig. 3:**
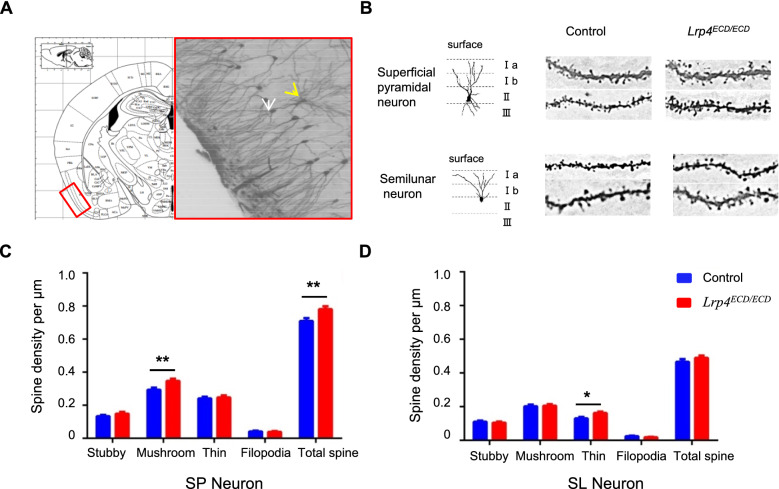
Increased spine density of superficial pyramidal neuron in *Lrp4*^*ECD/ECD*^ mice. **A** Representative Golgi staining of piriform cortical neurons in mice. **B** The representative spine on mice’s superficial pyramidal neuron (SP, arrowhead in **A**) and semilunar neuron (SL, arrow in **A**). **C** Quantification of spine density on SP neurons. The mature and total spine density of SP neurons in *Lrp4*^*ECD/ECD*^ mice were more than in the control mice. **D** Quantification of spine density on SL neurons. *Lrp4*^*ECD/ECD*^ mice exhibited a similar spine density of SL neurons than the control mice, except for a little increase in the thin type of spine. (Control mice, n = 6; *Lrp4*^*ECD/ECD*^ mice, n = 6. Values were means ± SEM. * P < 0.05, **P < 0.01)

To explore whether the morphology of piriform cortical neurons in *Lrp4*^*ECD/ECD*^ mice changed or not, we used Golgi staining to observe the neuronal dendritic spines in the *Lrp4*^*ECD/ECD*^ mice, compared with littermate control mice (Fig. 3). There were two different neurons in the second layer of the piriform cortex (Fig. 3B). One type was semilunar (SL) cell lacking basal dendrites, and the other was superficial pyramidal (SP) cell with both apical dendrites and basal dendrites. The mature spines (mushroom type) and total spine density on SP neurons were increased in the piriform cortex of *Lrp4*^*ECD/ECD*^ mice (Fig. 3C), which implied a potential increase in functional synaptic transmission. Except for a little rising of the thin type of spine in SL neurons, other types of spine density in *Lrp4*^*ECD/ECD*^ mice exhibited similar to the control mice (Fig. 3D).

### Enhanced EPSC of the piriform cortical neurons in *Lrp4*^*ECD/ECD*^ mice

**Fig. 4:**
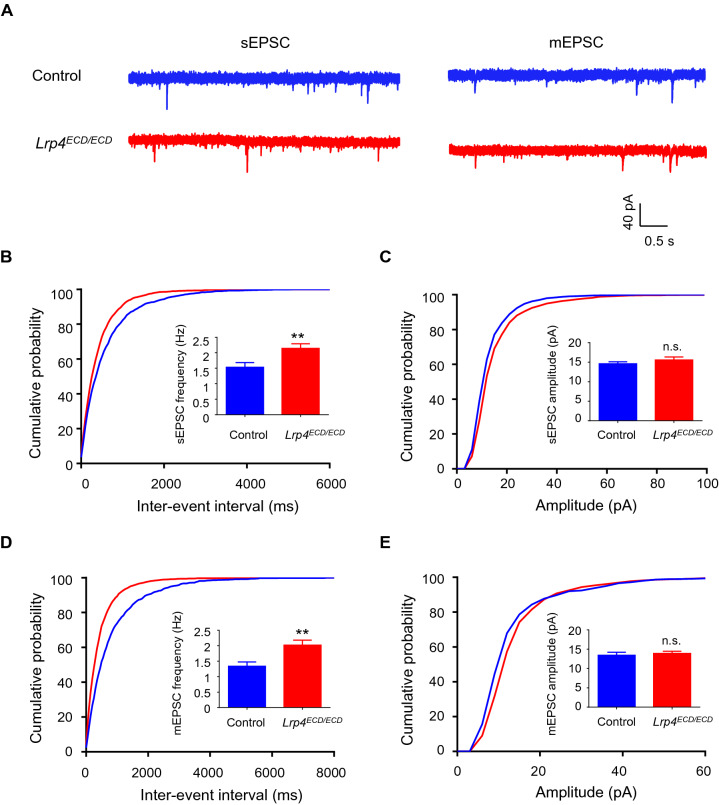
Enhanced excitatory synaptic transmission of the piriform cortical neurons in *Lrp4*^*ECD/ECD*^ mice. **A** Representative recording trace of sEPSC and mEPSC in the piriform cortex. **B** Frequency of spontaneous excitatory postsynaptic current (sEPSC) of the piriform cortical neurons in *Lrp4*^*ECD/ECD*^ mice increased. **C** The amplitude of the piriform cortical neuronals EPSC in *Lrp4*^*ECD/ECD*^ mice was similar to that in the control mice (control mice, n = 4, neurons, n = 18; *Lrp4*^*ECD/ECD*^ mice, n = 3; neurons, n = 15). **D** Frequency of miniature excitatory postsynaptic current (mEPSC) of the piriform cortical neurons in *Lrp4*^*ECD/ECD*^ mice elevated. **E** The amplitude of the piriform cortical neuronal mEPSC in *Lrp4*^*ECD/ECD*^ mice was no different from the control mice (control mice, n = 5; neurons, n = 16; *Lrp4*^*ECD/ECD*^ mice, n = 6; neurons, n = 18). (Values were means ± SEM. n.s., no significant; **P < 0.01)

In whole-cell patch-clamp configuration, piriform cortex pyramidal neurons were recorded in mice (Fig. 4). Compared with the control mice, *Lrp4*^*ECD/ECD*^ mice exhibit a high frequency of sEPSC (Fig. 4B) and mEPSC (Fig. 4D) in the piriform cortical neurons. No change was observed in the amplitude of mEPSC (Fig. 4C) and sEPSC (Fig. 4E). The results suggested hyperfunction of glutamatergic transmission in *Lrp4*^*ECD/ECD*^ mice, consistent with increased spine density (Fig. 3).

### Impaired olfactory function in both *Lrp4*^*ECD/ECD*^ mice and *Lrp4* cKO mice

**Fig. 5:**
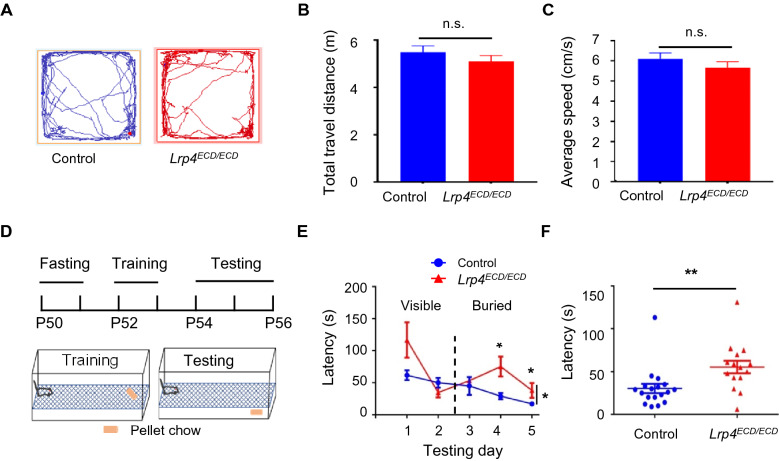
The impaired olfactory function of *Lrp4*^*ECD/ECD*^ mice. **A** Representative trace of mice in the open-field test. **B**, **C** There was no difference between *Lrp4*^*ECD/ECD*^ mice and the control mice in the total travel distance (**B**) and average speed (**C**) (control mice, n = 10; *Lrp4*^*ECD/ECD*^ mice, n = 10). **D** Schematic diagram of buried food-seeking test. Food deprived for 2 days before the training, training 2 days, and testing 3 days; mice were free to access enough water all the time; food was visible in training trials and buried in testing trials. **E** *Lrp4*^*ECD/ECD*^ mice spent more time finding the buried pellet chow than control mice. **F** The latency to find pellet chow in the testing days. (Control mice, n = 6; *Lrp4*^*ECD/ECD*^ mice, n = 5. Values were means ± SEM. n.s., no significant; *P < 0.05, **P < 0.01)

Both spine density and electrophysiology of piriform cortical neurons were changed in *Lrp4*^*ECD/ECD*^ mice. In order to explore the function of LRP4 in the olfactory pathway, a buried food-seeking test (Fig. 5) was performed. Firstly, in the open-field test, we found that the locomotive ability of *Lrp4*^*ECD/ECD*^ mice was not affected (Fig. 5A), the total travel distance (Fig. 5B) and average speed (Fig. 5C) were not changed. Then, the buried food-seeking test was conducted. The mice were food-deprived for 2 d before training 3 d, and testing was started (Fig. 5D). Mice were free to access enough water all the time. *Lrp4*^*ECD/ECD*^ mice spent more time finding the buried pellet chow in the test trials than control mice (Fig. 5E, F), suggesting that olfactory function may be impaired.

**Fig. 6:**
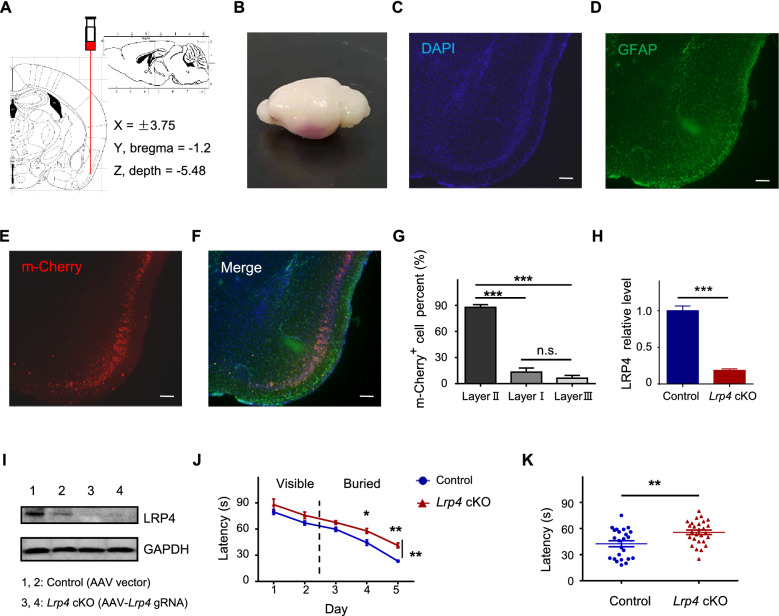
The impaired olfactory function of *Lrp4* cKO mice in the piriform cortex. **A** Representative injection trace of AAV into CAG-*Cas9* mice in the piriform cortex. **B** The representative brain of AAV infection, 21d after stereotactic injecting AAV into the piriform cortex of CAG-*Cas9* mice. **C**–**F** Immunofluorescence staining showed AAV virus infection with layer II cells in the piriform cortex. **G** mCherry positive cell percent in the piriform cortex (AAV injection CAG-*Cas9* mice n = 4). **H**, **I** *Lrp4* was knockout in the piriform cortex of *Lrp4* cKO mice. Piriform cortex was used for western blotting (**I**) and quantification of LRP4 level relative to GAPDH (**H**). LRP4 relative level in control mice was normalized to a value of 1. **J**, **K** *Lrp4* cKO mice spent more time finding the buried food than control mice. (*Lrp4* cKO mice, CAG-*Cas9* male mice injected with AAV-*Lrp4* gRNA, n = 9; control mice, CAG-*Cas9* male mice injected with AAV vector, n = 8. Scar bar = 15 μm. Values were means ± SEM. n.s., no significant; *P < 0.05, **P < 0.01, ***P < 0.001)

Besides, *Lrp4* cKO mice, via injecting AAV-*Lrp4* gRNA into the piriform cortex of CAG-*Cas9* mice, were engaged in evaluating the olfactory function (Fig. 6). Immunofluorescence staining results showed that Layer II cells infected with AVV appeared red in the piriform cortex region (Fig. 6E, F). The layer II cells of the piriform cortex had higher virus infection compared with layer I and layer III (Fig. 6G). *Lrp4* was effectively knockout in the piriform cortex of *Lrp4* cKO mice (Fig. 6H, I). Moreover, the time was prolonged for searching buried food in *Lrp4* cKO mice (Fig. 6J, K).

In conclusion, we reported that *Lrp4* was highly expressed in layer II of the piriform cortex, and piriform cortical neurons in *Lrp4*^*ECD/ECD*^ mice exhibited an increase in mature spine density and enhanced both sEPSC and mEPSC. Moreover, impairment of olfactory function was found in both *Lrp4*^*ECD/ECD*^ mice and *Lrp4* cKO mice, suggesting the non-negligible role of the full length of LRP4 in the piriform cortex. These results implied that the full length of LRP4 in the piriform cortex was necessary to maintain synaptic plasticity and the integrity of the olfactory signal transmission.

## Discussion

The piriform cortex, a densely packed-cell-body layer, exhibits highly structural plasticity, such as dendritic remodeling, spine genesis and synaptic reorganization [30], and the synaptic plasticity in the piriform cortex encodes olfactory information, associative memory and sensory processing [27–30]. As a higher olfactory center and the largest area of the olfactory cortex, the piriform cortex receives direct input from the olfactory bulb and is connected with all of the entorhinal cortical domains [31, 32]. Similar to the hippocampus being an evolutionally conserved paleocortex, the piriform cortex is also a phylogenetically ancient structure [30, 33].

Astrocytes, the most abundant cell type in the brain [34, 35], have a star-like morphology. The cell soma radiates many branches, and the primary branches gradually divide into finer and finer processes to form a dense network [34], which is more complex than immunostaining. In our immunostaining result, GFAP positive cells did not have the astrocyte characteristic morphology. The dentate gyrus is a particular region of the dense granular cells, and astrocytes cannot be discriminated in the condensed neuron layer. Herein, the piriform cortex cannot efficiently stain the astrocyte.

Layer II of the piriform cortex is developed prenatally and is regarded as devoid of postnatally proliferative capacity before. However, a subpopulation of immature neurons in layer II express DCX (doublecortin) and PSA-NCAM (polysialylated neural cell adhesion molecule) [30, 36–38]. Therefore, the piriform cortex possesses slight adult neurogenesis [38–40]. So, PSA-NCAM positive neurons may also express GFAP, and the GFAP-positive cells in the piriform cortex may not be astrocytes [41]. Zhang et al*.* found that *Lrp4* is indispensable in adult neurogenesis [52], which might bring the complicated effect of LRP4 in the piriform cortex.

The olfactory sensory neurons locate in the olfactory mucosa, bulb, and olfactory cortex [42, 43]. The olfactory cortex integrates the olfactory signals, forms olfactory memories, and integrates specific olfactory signals with sensory information, such as color, taste, shape, and spatial location [44]. Complex odor signal analysis and integration rely on higher-level central structures, such as the piriform cortex [45]. The olfactory signal analysis in the piriform cortex relates to specific olfactory memory and involves olfactory sensitization and passivation [46]. Although the recognition mechanism of odor molecules has been deeply understood in the past ten years [47], it is still unclear how to complete the integration and modulation of olfactory signals in the high cortex. Here, LRP4 was highly expressed in the piriform cortex, and *Lrp4*^*ECD/ECD*^ mice exhibited high spine density and high frequency of sEPSC and mEPSC in the piriform cortical neurons, which indicated LRP4 might regulate the transmission of olfactory signals.

In the buried food-seeking test, results showed that the time to find food was significantly longer in *Lrp4*^*ECD/ECD*^ mice than in the control mice. The test results suggested that *Lrp4*^*ECD/ECD*^ mice may have olfactory dysregulation. Moreover, similar results were seen in *Lrp4* cKO mice. Strikingly, *Lrp4*^*ECD/ECD*^ mice showed reduced body weight and brain weight, suggesting that LRP4 affected the development. The previous report also found that *Lrp4*^*ECD/ECD*^ mice showed typical tight-knit morphology [6]. This kind of limb clinging to the tail after the tail’s suspension also appeared in the neurological disease model mice, suggesting that the brain’s neurological function in the *Lrp4*^*ECD/ECD*^ mice may be impaired.

Kariminejad et al*.* reported one case in which a patient identified the novel homozygous mutation c.289G > T in *Lrp4* exon 3. This nucleotide exchange leads to a premature stop codon at amino acid 97 (p.E97X) at the beginning of the large extracellular domain. The patient had mixed-type hearing loss, vertebral anomalies, and renal hypoplasia [48]. Another study reported a novel splice variant in *Lrp4* (c.316t1G > A), and the missense variant adds 29 non-native amino acids with premature stop-codon, which causes the *Lrp4* encoding to terminate prematurely. The patient had short feet, frontal bossing, and other symptoms [49]. These findings suggest that the ECD of LRP4 plays a vital role in limb development, kidney development, and brain development. The results imply that the full length of LRP4 was non-negligible in the development.

Wnt signaling regulates brain development and synapse maturation [50]. LRP4 has an antagonistic function on LRP6-mediated Wnt/β-catenin activation. Ramos-Fernández et al*.* reported that Wnt-7a stimulates dendritic spine formation in the hippocampus via inhibiting GSK-3β (glycogen synthase kinase-3β), triggering TCF/LEF-dependent gene transcription and promoting *PSD-95* expression [51]. Our results showed that the dendritic spine density in the piriform cortex of *Lrp4*^*ECD/ECD*^ mice significantly increased. Therefore, we hypothesized that the ECD of LRP4 might promote dendritic spine formation. SP dendritic mature spine in the piriform cortex increased significantly, related to neuronal physiological functions via participating in different neural circuits.

Excitatory synaptic transmission of the piriform cortical neurons in *Lrp4*^*ECD/ECD*^ mice was enhanced. Pohlkamp et al*.* examined synaptic function in the *Lrp4*^*ECD/ECD*^ mice by recording LTP in CA3-CA1 Schaeffer collaterals [7]. CA3-CA1 projections are a classic model for measuring and understanding synaptic plasticity. There is a substantial deficit in late-phase LTP in *Lrp4*^*ECD/ECD*^ mice [7], implying the loss of the ICD and TMD may severely impair the LRP4 function. Sun et al*.* found that conditional knockout of *Lrp4* in astrocytes suppresses glutamatergic transmission in the CNS [1]. The frequency of sEPSC and mEPSC in hippocampal CA1 pyramidal neurons was reduced, and synaptic plasticity was also impaired in *Lrp4* conditioned knockout mice [1, 6]. sEPSC and mEPSC frequency in piriform cortical neurons were enhanced in *Lrp4*^*ECD/ECD*^ mice. Though the pyramidal cell type recorded for electrophysiology was not clear, the enhancement of EPSCs was consistent with increasing the piriform cortex’s dendritic spine density. The inconsistencies were that ECD of LRP4 remained in piriform cortical neurons in this research, but in previous literature, *Lrp4* was conditioned knockout in astrocytes [1, 6]. Moreover, LRP4 is indispensable in adult neurogenesis [52]. We speculated that excitatory neurotransmitters might be impaired in the *Lrp4* cKO mice leading to an abnormal behavior similar to *Lrp4*^*ECD/ECD*^ mice, but the mechanism might be complicated.

## Conclusions

In conclusion, our results showed that *Lrp4* was highly expressed in layer II of the piriform cortex. In *Lrp4*^*ECD/ECD*^ mice, piriform cortical neuronal dendritic mature spine density increased, and both sEPSC and mEPSC were enhanced. Moreover, the olfactory function was impaired in *Lrp4*^*ECD/ECD*^ mice and *Lrp4* cKO mice. These results suggest that the full length of LRP4 in the piriform cortex was necessary to maintain synaptic plasticity and the olfactory signal transmission pathway. The molecular regulating mechanism needs further exploration.

## Supplementary Information

**Additional file 1: Fig S1. **Lower body and brain weight of *Lrp4*^*ECD/ECD*^ mice. **A** Representative images of one-month-old *Lrp4*^*ECD/ECD*^ mice compared with the control mice. *Lrp4*^*ECD/ECD*^ mice were smaller than control mice. **B** *Lrp4*^*ECD/ECD*^ mice’s body weight was significantly lower, compared to the control mice (control mice, n = 16; *Lrp4*^*ECD/ECD*^ mice, n = 12). **C** Representative brain images of *Lrp4*^*ECD/ECD*^ mice and the control mice. **D** *Lrp4*^*ECD/ECD*^ adult mice’s brain weight was lower, compared with the control mice (control mice, n = 12; *Lrp4*^*ECD/ECD*^ mice, n = 12). **E** *Lrp4*^*ECD/ECD*^ mice showed typical tight-knit morphology. (Values were means ± SEM.* P < 0.05, ** P < 0.01).

## Data Availability

The datasets used or analyzed in our study are available from the corresponding author on reasonable request.

## Abbreviations

AD: Alzheimer’s disease
β-gal: β-Galactosidase
cKO: Conditional knockout
CNS: Central nervous system
DCX: Doublecortin
ECD: Extracellular domain
ICD: Intracellular domain
LRP4: Low-density lipoprotein receptor-related protein 4
mEPSC: miniature excitatory postsynaptic current
NMJ: Neuromuscular junction
PBS: Phosphate-buffered sodium
PEI: Polyethylenimine
PFA: Paraformaldehyde
PSA-NCAM: Polysialylated neural cell adhesion molecule
sEPSC: Spontaneous excitatory postsynaptic current
TMD: Transmembrane domain
X-gal: 5-Bromo-4-chloro-3-indolyl-beta-d-galacto-pyranoside
