# Supplementary material for: LRP4 is required for the olfactory association task in the piriform cortex
Source: Cell Biosci. 2022 May 7;12:54. doi: 10.1186/s13578-022-00792-9 (PMC9080164; doi:10.1186/s13578-022-00792-9)
Supplement: Supplementary file 1 — Additional file 1: Fig S1. Lower body and brain weight of Lrp4ECD/ECD mice. A Representative images of one-month-old Lrp4ECD/ECD mice compared with the control mice. Lrp4ECD/ECD mice were smaller than control mice. B Lrp4ECD/ECD mice’s body weight was significantly lower, compared to the control mice (control mice, n = 16; Lrp4ECD/ECD mice, n = 12). C Representative brain images of Lrp4ECD/ECD mice and the control mice. D Lrp4ECD/ECD adult mice’s brain weight was lower, compared with the control mice (control mice, n = 12; Lrp4ECD/ECD mice, n = 12). E Lrp4ECD/ECD mice showed typical tight-knit morphology. (Values were means ± SEM.* P < 0.05, ** P < 0.01). [file 13578_2022_792_MOESM1_ESM.pptx]

## Slide 1
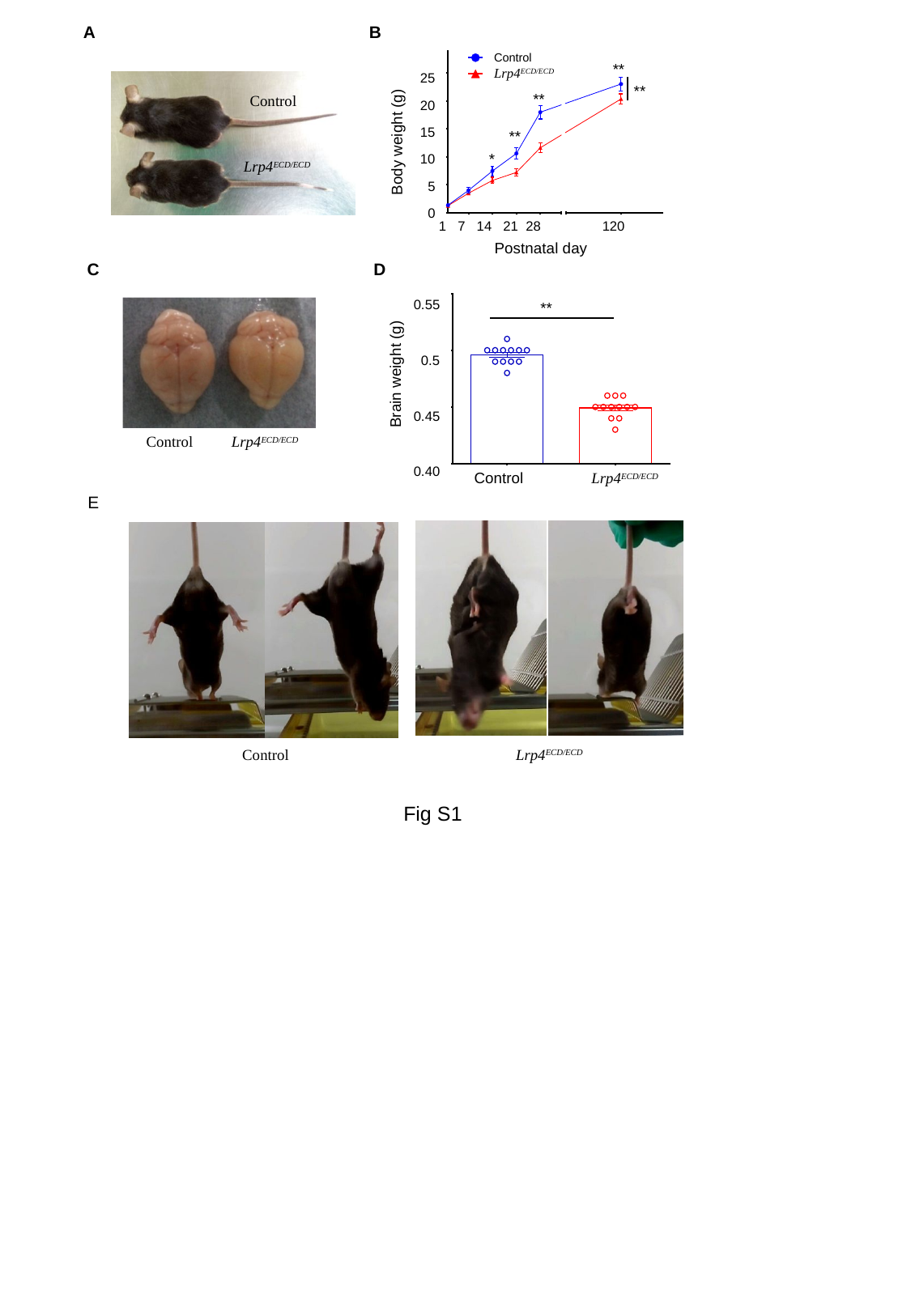

A B
Control
Lrp4ECD/ECD
25
20
15
10
5
0
**
**
**
Control
**
Body weight (g)
*
Lrp4ECD/ECD
1 7 14 21 28 120
Postnatal day
0.55
0.5
0.45
0.40
C D
**
Brain weight (g)
Control Lrp4ECD/ECD
Control Lrp4ECD/ECD
E
Control Lrp4ECD/ECD
Fig S1
